# Association Between *Giardia* Genotype and Oxidative Stress Biomarkers Among *Giardia*-Infected Children: A Case–Control Study

**DOI:** 10.1007/s11686-022-00548-y

**Published:** 2022-05-10

**Authors:** Amira Ismail, Aida A. Abdel-Magied, Abeer A. Elhenawy, Hala A. El-Nahas

**Affiliations:** Department of Medical Parasitology, Faculty of Medicine, Mansoura University, Mansoura, 35516 Egypt

**Keywords:** *Giardia*, Biomarkers, Oxidative stress, Immunosuppressive, Genotype

## Abstract

**Purpose:**

*Giardia duodenalis* is the most common worldwide intestinal protozoal infection. The implication of free radicals in organ injury occurs through oxidative stress. Infections as *Giardia* may act as a triggering or promoting factor for oxidative stress, particularly in children with compromised immunity. Besides, the effect of *Giardia* genotype on oxidative stress status is yet to be explored. Therefore, we sought to compare the oxidative stress status between *Giardia* positive cases (case group) and *Giardia* negative cases (control group), and to explore the association between *Giardia* genotype and the level of oxidative stress markers in *Giardia*-infected children, especially those receiving immunosuppressive therapy.

**Methods:**

Pediatric patients attending Mansoura University Children Hospital in the period from April 2015 to October 2016 were enrolled. Both case (*n* = 50) and control (*n* = 50) groups were further subdivided into immunosuppressive therapy recipients (ITR) and non-immunosuppressive therapy recipients (NITR). Genotyping of *Giardia* from positive stool samples by PCR was carried out, and oxidative stress markers were measured from venous blood samples.

**Results:**

*Giardia* positive cases had higher levels of Malondialdehyde (MDA) and lower levels of total antioxidant capacity (TAC). MDA highest level was associated with mixed genotypes A and B, while the highest TAC level was associated with *Giardia* genotype A in both ITR and NITR cases.

**Conclusion:**

Elevated oxidative stress biomarkers in pediatric patients infected with specific *Giardia* genotypes should receive considerable attention, because if prompt treatment is not conducted, oxidative damage may occur in patients with giardiasis, especially those receiving immunosuppressive therapy.

## Introduction

*Giardia duodenalis *is a common gastrointestinal protozoan parasite, causing diarrheal illness in humans worldwide [1]. In developing countries, giardiasis is a significant public health concern, particularly in children under 5 years of age [2].

Although the variability in infection and disease is well known, the cause(s) of this variability are largely unknown [3]. Both host and parasite factors contribute to the pathogenesis of giardiasis, and ongoing research in this field may elucidate genotype/assemblage-specific pathogenic mechanisms [4].

Host factors as redox regulation refers to the set of regulatory processes controlled by redox signaling. This process protects the body against oxidative damage, restores the original state of “redox homeostasis” after temporary exposure to free radicals, and keeps them at a steady state by maintaining the equilibrium between their release and elimination rates, thus protecting the body from the injurious effect and organ damage of oxidative stress [5, 6].

Regarding parasite factors, Nash *et al.* [7] demonstrated differences in infectivity and virulence among isolates. In some studies, genotype A was less virulent than genotype B [7], while an association between assemblage AII and symptomatic infections, and between assemblage B and asymptomatic infections was proved in the other studies [8]. These contradictory results make data on virulence inconclusive [3]. *Giardia* assemblages may not be solely responsible for the presence of clinical manifestations, but also host factors may be involved, as well [8].

Both host and parasite factors together with their interactions affect metabolism and hence the nutritional status of the host worsening nutritional status of immunocompromised or malnourished patients. A reciprocal relationship between infection and malnutrition exists. Malnourished individuals’ response to giardiasis results in free radical release as oxidative stress agents that lead to changes in lipoprotein composition and altered metabolism [9]. In malnourished or immunosuppressed hosts, immunosuppression has different consequences on the host depending on its magnitude and will alter the range of pathogens they are susceptible to and may increase their virulence [10].

Therefore, we sought to compare the oxidative stress status as a measure of redox state between *Giardia* positive cases (case group) and *Giardia* negative cases (control group), and to explore the association between *Giardia* genotype and the level of oxidative stress biomarkers in *Giardia*-infected children receiving immunosuppressive therapy as compared with non-immunosuppressive therapy recipients.

## Materials and Methods

This is a case–control study that was conducted at Mansoura University Children Hospital and Medical Parasitology Department, Faculty of Medicine, Mansoura University in Egypt. Pediatric patients attending outpatient clinics at Mansoura University Children Hospital in the period from April 2015 through October 2016 were enrolled. A total of 1260 stool samples were examined microscopically for *Giardia* infection to detect *Giardia* positivity rate. An informed written consent was obtained from parents or guardians of children.

The case group included 50 pediatric patients with the following inclusion criteria: (1) age (1–10 years), (2) either sex, (3) complaining of gastrointestinal symptoms such as abdominal pain, diarrhea, or flatulence, and (4) positive for *Giardia* by direct stool examination. Those who have other parasitic or bacterial infections were excluded from the study. The case group was further classified into two sub-groups: (a) non-immunosuppressive therapy recipients (NITR) (*n* = 25): those who had only symptoms of gastroenteritis and (b) immunosuppressive therapy recipients (ITR) (*n* = 25): those receiving immunosuppressive therapy for any cause and with symptoms of gastroenteritis. The control group included 50 age- and sex-matched children, negative for *Giardia* by direct stool examination, and free of any other parasitic or bacterial infection to compare oxidative stress status. The control group was similarly classified into two sub-groups; (a) NITR controls (*n* = 25): healthy children, free of any gastrointestinal complaints; referred children to the pediatric outpatient clinic for a routine checkup with normal physical examination and laboratory results and (b) ITR controls (*n* = 25): those receiving immunosuppressive therapy for any cause but free of giardiasis.

### Stool Samples

Three consecutive stool samples on each other day were collected from each participant in the study. Stool specimens were collected in dry, sterile, labeled, wide-mouthed plastic containers with tight-fitting cover. The samples were not contaminated with urine and transported to the laboratory for immediate microscopic examination using the direct wet smear and formalin–ether sedimentation methods. Stool samples from cases and controls were stained by modified acid-fast for *Cryptosporidium* spp., *Cyclospora* and *Cystoisospora*, and were tested for common bacterial pathogens using standard culture methods to exclude other parasitic and bacterial infection. Three consecutive stool samples were examined before ruling out *Giardia* infection in the control group. Then, QIAamp DNA Stool Mini Kit (QIAGEN Sample and Assay Technologies, Hilden, Germany) Catalog no. 51504 was used for *Giardia* DNA extraction from microscopically positive stool samples. Then, PCR was employed to identify *Giardia* genotypes in *Giardia* positive cases using two sets of primers for detection of *Giardia lamblia* assemblages A and B designed against the coding region of the *tpi* gene [11]. The Primers used for assemblage A amplification were forward primer (A-for) 5/-GGAGACCGACGAGCAAAGC-3/ (positions 839 to 857 on the WB sequence) and reverse primer (A-rev) 5/-CTTGCCAAGCGCCTCAA-3/ (positions 970 to 986 on the WB sequence). The primers used for assemblage B amplification were: forward primer (B-for) 5/-AATAGCAGCACA RAACGTGTATCTG-3/ (positions 126 to 150 on the BAH-12 sequence) and reverse primer (B-rev) 5/-CCCATGTCCAGCAGCATCT-3/ (positions 188 to 206 on the BAH-12 sequence) [11]. A 148-bp fragment of the assemblage A gene was amplified with primers A-for and A-rev (A-PCR). An 81-bp fragment of assemblage B gene was obtained with primers B-for and B-rev (B-PCR). Finally, PCR products were separated by agarose gel electrophoresis.

### Blood Samples

Venous blood samples (5 ml) were collected from all cases and controls. Sera were separated and stored at − 80 °C for estimation of oxidative stress biomarkers. Kits purchased from Biodiagnostic, Dokki-Giza-Egypt (Malondialdehyde MDA), lipid peroxide assay (CAT. NO. MD 25 29), and total antioxidant capacity, TAC assay: (CAT. NO. TA 25 13) were utilized according to the manufacturer’s instructions. Samples were read by Automatic Biochemistry Analyzer (RBK spectra S. No A5150315 RIE).

### Statistical Analysis

The collected data were coded, processed, and analyzed using the Statistical Package of Social Science (SPSS) program for Windows (Standard version 22). The normality of data was first tested with one-sample Kolmogorov–Smirnov test. Qualitative data were described using numbers and percentages. Association between categorical variables was tested using Chi-square test. Continuous variables were presented as mean ± SD (standard deviation). Student’s *t* test was used to compare two means, while ANOVA test was used to compare more than two means. Pearson correlation was used for continuous data.

## Results

**Fig.1:**
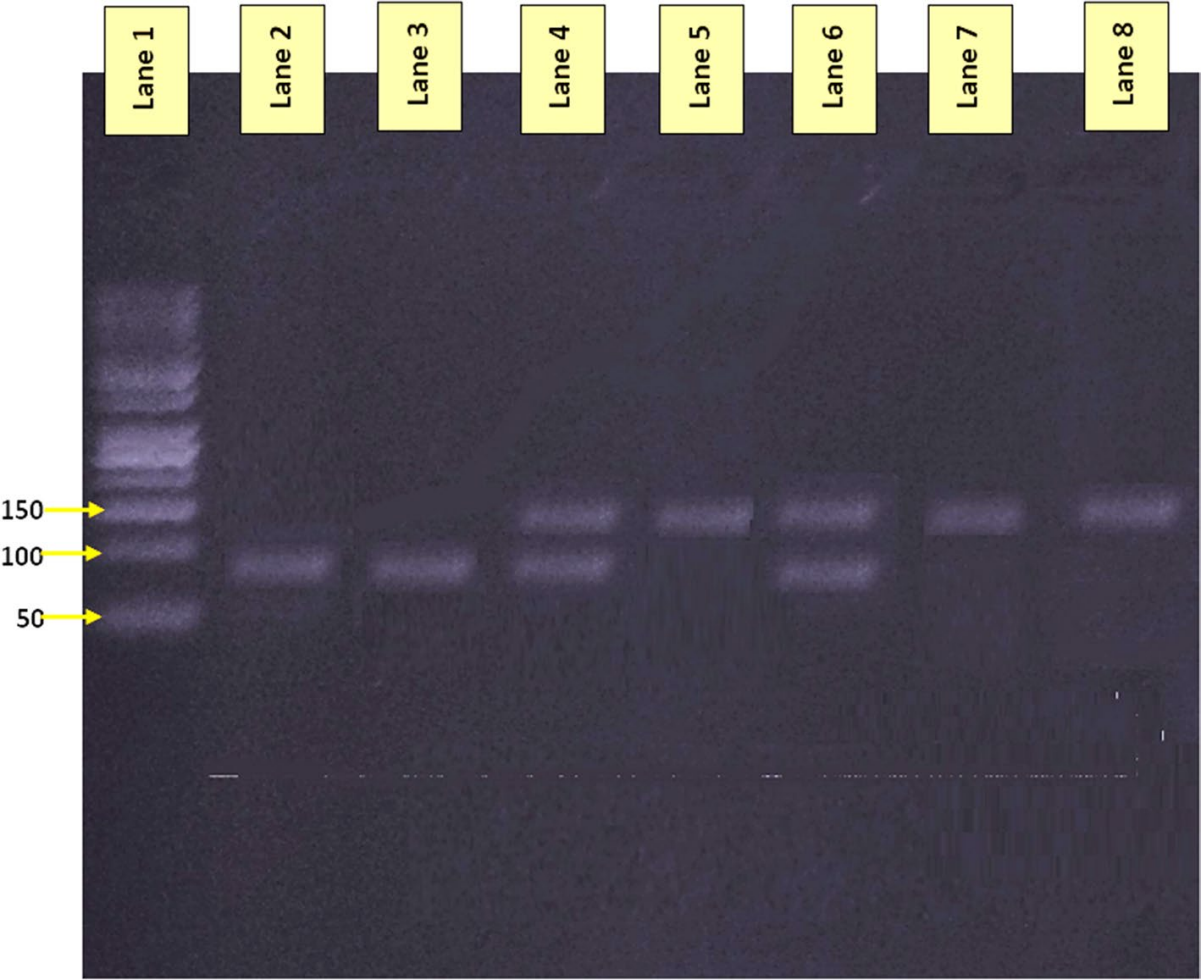
*Giardia* genotypes separated on agarose gel electrophoresis. Lane 1 represents 50-bp DNA marker; *Giardia* assemblage A, 148-bp product (lanes 5, 7 and 8); *Giardia* assemblage B, 81-bp product (lanes 2 and 3); both A and B *Giardia* assemblages (lanes 4 and 6)

The prevalence of *Giardia* among participants detected by direct stool examination was 20.23% (255/1260). *Giardia* genotype A was the most prevalent genotype (42%), followed by genotype B (34%), while mixed infections by both genotypes were 24%. Figure 1 shows the results of *Giardia* genotyping by PCR amplification of *tpi* gene.

There was no statistically significant difference between ITR and NITR groups regarding the *Giardia* genotype (Table 1).

**Table 1:** Comparison between NITR cases and ITR cases regarding *Giardia* genotype

Genotype	NITR case (*n* = 25)	ITR cases (*n* = 25)	*χ*^2^	*p* Value
A	12 (48%)	9 (36%)	0.739	0.390
B	8 (32%)	9 (36%)	0.089	0.765
AB	5 (20%)	7 (28%)	0.439	0.508

*χ*^2^ Chi-square test

There were statistically significant differences between each two groups and sub-groups. Regarding the level of MDA, the highest level of MDA was generally higher in cases than controls; highest in ITR cases followed by ITR controls, then in NITR cases followed by NITR controls (Table 2). On the contrary, the highest TAC level was in NITR controls followed by NITR cases, and then ITR controls followed by ITR cases (Table 3).

**Table 2:** MDA level comparison across sub-groups

MDA	Mean ± SD	Min–max	*t* test	*p* Value
Cases (*n* = 50)	37.68 ± 16.51	20–80	3.853	< 0.001**
Controls (*n* = 50)	26.96 ± 10.68	17–60		
NITR cases (*n* = 25)	27.40 ± 5.80	20–40	7.326	< 0.001**
NITR controls (*n* = 25)	18.76 ± 1.05	17–20		
ITR cases (*n* = 25)	47.96 ± 17.41	34–80	3.220	0.002*
ITR controls (*n* = 25)	35.16 ± 9.59	25–60		
NITR cases (*n* = 25)	27.40 ± 5.80	20–40	5.603	< 0.001**
ITR cases (*n* = 25)	47.96 ± 17.41	34–80		
NITR controls (*n* = 25)	18.76 ± 1.05	17–20	8.496	< 0.001**
ITR controls (*n* = 25)	35.16 ± 9.59	25–60		

*Statistical significance

**High statistical significance

**Table 3:** TAC level comparison across sub-groups

TAC	Mean ± SD	Min–max	*t* test	*p* Value
Cases (*n* = 50)	1.44 ± 0.27	0.9–1.9	4.523	< 0.001**
Controls (*n* = 50)	1.702 ± 0.31	1.1–2.1		
NITR cases (*n* = 25)	1.64 ± 0.18	1.3–1.9	9.068	< 0.001**
NITR cases (*n* = 25)	1.99 ± 0.067	1.9–2.1		
ITR cases (*n* = 25)	1.24 ± 0.18	0.9–1.4	3.652	0.001*
ITR controls (*n* = 25)	1.42 ± 0.15	1.1–1.6		
NITR cases (*n* = 25)	1.64 ± 0.18	1.3–1.9	7.58	< 0.001**
ITR cases (*n* = 25)	1.24 ± 0.18	0.9–1.4		
NITR controls (*n* = 25)	1.99 ± 0.067	1.9–2.1	17.24	< 0.001**
ITR controls (*n* = 25)	1.42 ± 0.15	1.1–1.6		

*Statistical significance

**High statistical significance

MDA level was related to *Giardia* genotype as the highest MDA level was associated with mixed genotypes A and B in both groups, while the lowest level was associated with genotype A in both groups (NITR cases and ITR cases) (Table 4). In comparison, there was a high statistically significant difference between *Giardia* genotype and TAC level across sub-groups. The highest TAC level was associated with *Giardia* genotype A and the lowest with genotype AB in either group (NITR cases and ITR cases) (Table 5).

**Table 4:** Relation between *Giardia* genotype and MDA levels among *Giardia* positive cases

MDA	*N*	Mean ± SD	Min–max	ANOVA test	*p* Value
*Giardia* positive cases (*n* = 50)					
Genotype A	21	28.24 ± 6.41^ab^	20–37	26.92	< 0.001**
Genotype B	17	34.76 ± 6.79^bc^	25–46		
Genotype AB	12	58.33 ± 20.59^ac^	30–80		
NITR cases (*n* = 25)					
Genotype A	12	23 ± 2.04^ab^	20–28	34.84	< 0.001**
Genotype B	8	28.62 ± 3.33^bc^	25–35		
Genotype AB	5	36 ± 4.18^ac^	30–40		
ITR cases (*n* = 25)					
Genotype A	9	35.22 ± 0.97^ab^	34–37	179.186	< 0.001**
Genotype B	9	40.22 ± 3.38^bc^	35–46		
Genotype AB	7	74.29 ± 7.31^ac^	65–80		

*Statistical significance

**High statistical significance

*N.B*. ab, bc, ac Each two similar letters indicate significance difference

**Table 5:** Relation between *Giardia* genotype and TAC levels among *Giardia* positive cases

TAC	*N*	Mean ± SD	Min–max	ANOVA test	*p* Value
*Giardia* positive cases (*n* = 50)					
Genotype A	21	1.62 ± 0.20^ab^	1.4–1.9	23.16	< 0.001**
Genotype B	17	1.42 ± 0.16^bc^	1.2–1.7		
Genotype AB	12	1.14 ± 0.23^ac^	0.9–1.5		
NITR cases (*n* = 25)					
Genotype A	12	1.79 ± 0.05^ab^	1.7–1.9	53.551	< 0.001**
Genotype B	8	1.56 ± 0.1^bc^	1.4–1.7		
Genotype AB	5	1.38 ± 0.08^ac^	1.3–1.5		
ITR cases (*n* = 25)					
Genotype A	9	1.40 ± 0.0^ab^	1.4 0–1.4	100.092	< 0.001**
Genotype B	9	1.28 ± 0.06^bc^	1.2–1.4		
Genotype AB	7	0.97 ± 0.09^ac^	0.9–1.1		

*Statistical significance

**High statistical significance

*N.B*. ab, bc, ac Every two similar letters indicate a significant difference

## Discussion

*Giardia duodenalis* is the most prevalent intestinal parasite worldwide, with about 280 million people suffering from symptomatic *Giardia* infection every year [12]**.** Although the distribution of *Giardia duodenalis* genotypes in humans has been increasingly reported in recent years, data on possible differences in pathogen transmission between age groups and virulence between genotypes are scarce [13]**.**

In the current study, *Giardia* positivity rate was 20.23% (255 positive cases out of 1260 stool samples) similar to Ismail* et al*. [14] who reported that *Giardia* prevalence was 18.9% in patients attending outpatient clinics of Cairo University hospitals.

In the present study, genotype A was the most prevalent (42%), followed by genotype B (34%), and then mixed infection with AB (24%). Either genotype A or B prevails in isolates from *Giardia* human studies conducted in different countries and on other continents, although contradictory results from various studies were obtained regarding their relative distributions [15]**.** In Turkey**,** Sonmez Tamer* et al*. [16] performed a pilot study for *G. duodenalis* genotyping in Kocaeli, Turkey. Assemblage A was identified in 50% of the total detected isolates, while assemblage B was identified in 31.8%, and assemblage AB was detected in 18.2% of the isolates, and this agreed with the results of the current study. In Africa, Assemblage B was the most predominant among typed samples in 18 out of 28 studies, while assemblage A was dominant in the remaining ten studies [17]. Differences in the prevalence of assemblages A and B might be attributed to the patients’ geographic locations included in our study and the different methods used in genotyping.

Regarding mixed infections, the reported prevalence is 2–21%, with more prevalence in underdeveloped countries [18]. In Egypt, the occurrence of mixed infection was found to be 15.4% in asymptomatic individuals and 16% in symptomatic individuals [19]. Although the genotypic separation of *G. intestinalis* genetic assemblages was relatively well recognized [20], the clinical or epidemiologic implication of *Giardia* infection with assemblage A versus B was poorly understood [21]. However, a previous study in Turkey reported that children infected with assemblage A were less associated with greater cyst shedding than children infected with assemblage B, promoting assemblage A spread [16] as observed in the present study. Regarding mixed infections, reported prevalence is 2–21%, with more prevalence in underdeveloped countries [18]. In Egypt, the occurrence of mixed infection was found to be 15.4% in asymptomatic individuals and 16% in symptomatic individuals [19].

To the best of our knowledge, there is no prior study investigating the correlation between *Giardia* genotype and the patients’ immune status. The present study investigated the relation between giardiasis and some oxidative stress biomarkers (MDA and TAC). There was a higher statistically significant difference between *Giardia* positive cases and controls regarding the serum level of MDA and TAC. The serum level of MDA was significantly higher in *Giardia* positive cases than the controls, while the serum level of TAC was significantly lower in *Giardia* positive cases than the controls. Regarding the comparison between immunosuppressive therapy recipients and non-immunosuppressive therapy recipients, we found that the highest MDA level was in ITR cases followed by ITR controls then NITR cases followed by NITR controls. Regarding TAC level, the highest level was in NITR controls followed by NITR cases, and then ITR controls followed by ITR cases. Additionally, there was a significant negative correlation between serum MDA and TAC levels among the studied groups.

Our results were in accordance with Abd Al-Wahab *et al.* [22], who found that the serum MDA levels for patients with various parasitic infections, including patients with giardiasis, were significantly higher than that in the control group.

On the other hand, Mona *et al.* [23] conducted a study to investigate the homeostasis of some micronutrients and antioxidants in human giardiasis. They found that *Giardia*-infected patients displayed levels of TAC, albumin, and bilirubin that were not significantly different from the controls.

These contradictory results may be related to some aspects of the immune response that provoke a state of oxidative stress in the host’s body [24]. The production of ROS might be significantly augmented in response to various pathophysiological conditions like inflammation, immunologic abnormalities, hypoxia, metabolism of drug, alcohol, therapeutic radiation, and deficiency in antioxidant vitamins, which often destroys cellular macromolecules (DNA, protein, and lipids) and other small antioxidant molecules [25].

The opportunistic nature of *G. duodenalis* was proposed and reported by de Lucio *et al.* [26] who found that several giardiasis cases in their study were immunocompromised patients including those with immunological abnormalities, cancer, diabetes, and HIV infections.

The severity of giardiasis might be assessed by the interaction between the parasite’s virulence, the immunological, nutritional status of the host, the presence of other co-pathogens, and the nature of intestinal microflora. Although different *G. duodenalis* genetic assemblages may produce various toxins or metabolic products that may participate in their pathogenicity, studies of the possible link between *G. duodenalis* genetic assemblages and virulence have thus far produced inconsistent results [27].

Previous human studies and experimental evidence revealed that *Giardia* infections with assemblage B might lead to pro-inflammatory intestinal responses. Several giardiasis patients infected with this assemblage were found to have microscopic duodenal inflammation and showed high fecal calprotectin levels [28]. Additionally, *in vivo* infections with *Giardia* assemblage B were shown to cause eosinophils’ intestinal recruitment [29].

Furthermore, reports had shown that *Giardia* infections with assemblage A might attenuate intestinal polymorph nuclear leucocytes chemotaxis [28]. Remarkably, *Giardia* assemblage A lowered infiltration of granulocytes, chemokines, and cytokines involved in the recruitment of neutrophils following intra-rectal installation of pro-inflammatory *Clostridium difficile* toxin; these effects were not observed with *in vivo Giardia* assemblage B infections [29].

Separating the role of the host’s immune response and the parasite’s role is essential to understand the disease variability. Many pathological mechanisms had been proposed based on *in vitro* experiments [30]**.** Host response against giardiasis associated with immune suppression may lead to oxidative stress that produces changes in lipoprotein composition, suggesting that the infection could influence the metabolic changes, worsening the nutritional status of infected patients [9].

## Conclusion

The present study showed that elevated levels of oxidative stress biomarkers in pediatric patients infected with specific *Giardia* genotypes should receive considerable attention, because if prompt treatment is not conducted, oxidative damage may occur in patients with giardiasis, especially those receiving immunosuppressive therapy.
